# Supplementary material for: Liquid Chromatography-Tandem Mass Spectrometry Method Development and Validation for the Determination of a New Mitochondrial Antioxidant in Mouse Liver and Cerebellum, Employing Advanced Chemometrics
Source: Molecules. 2025 Apr 24;30(9):1900. doi: 10.3390/molecules30091900 (PMC12074180; doi:10.3390/molecules30091900)
Supplement: Supplementary file 1 [file molecules-30-01900-s001.zip › molecules-3584383-supplementary.pdf]

Supplementary Material

Liquid Chromatography-Tandem Mass Spectrometry method development and validation for the determination of a new mitochondrial antioxidant in mouse liver and cerebellum, employing advanced chemometrics

Anthi Panara *[1], Dimitra Biliraki [2], Markus Nussbaumer [3,4], Michaela D. Filiou[3,4], Nikolaos S. Thomaidis [1] , Ioannis K. Kostakis [2], Evagelos Gikas [1]

Tables

Table S 1: %Percentage of each microspecies at different pH values 3
Table S 2: Log D values obtained for different pH... 6
Table S 3:Equations of the fortified curves of the liver samples 7
Table S 4: Equations of the fortified curves of the cerebellum samples 7
Table S 5: Results of accuracy (precision and trueness) in 5 fortification levels for cerebellum samples. 8

Table of figures

Figure S 1: MS/MS spectrum of TPP-HT and the proposed structures of the product ions. 2
Figure S 2: Proposed MS/MS Fragmentation mechanism of TPP-HT 2
Figure S 3: Pka diagram of TPP-HT ionized microspecies as a function of pH. The orange curve corresponds to structure 1, the green curve to structure 2, the blue curve to structure 3, and the red curve to structure 4. On the left side of the panel, the structures of ionic microspecies corresponding to the four curves can be found...3
Figure S 4: logD diagram as a function of pH of the most abundant microspecies of TPP-HT under the selected experimental conditions. On the left side of the panel, the structure of the microspecies is illustrated. 5
Figure S 5: Illustration of TPP-HT 7
Figure S 6: (a) QQ plot of fortified curves. (b) Homoscedasticity plot of the fortified curves for cerebellum samples. The QQ plots show that the residuals are normally distributed, and the homoscedasticity plots demonstrate the homoscedasticity of the residuals. 8
Figure S 7: (a) Fortified calibration curves of the samples of the 1st cluster. (b) Fortified calibration curves of the samples of the 2nd cluster. (c) One fortified curve fitting to the data of the 1st cluster. (d) One fortified curve fitting to the data of the 2nd cluster. 9

Optimization of mass spectrometric conditions

The MS/MS spectrum derived from the fragmentation of the precursor ion of TPP-HT is presented in Figure S1, alongside the proposed structures of the product ions generated from the CID procedure. The m/z values highlighted in green circles correspond to the product ions.

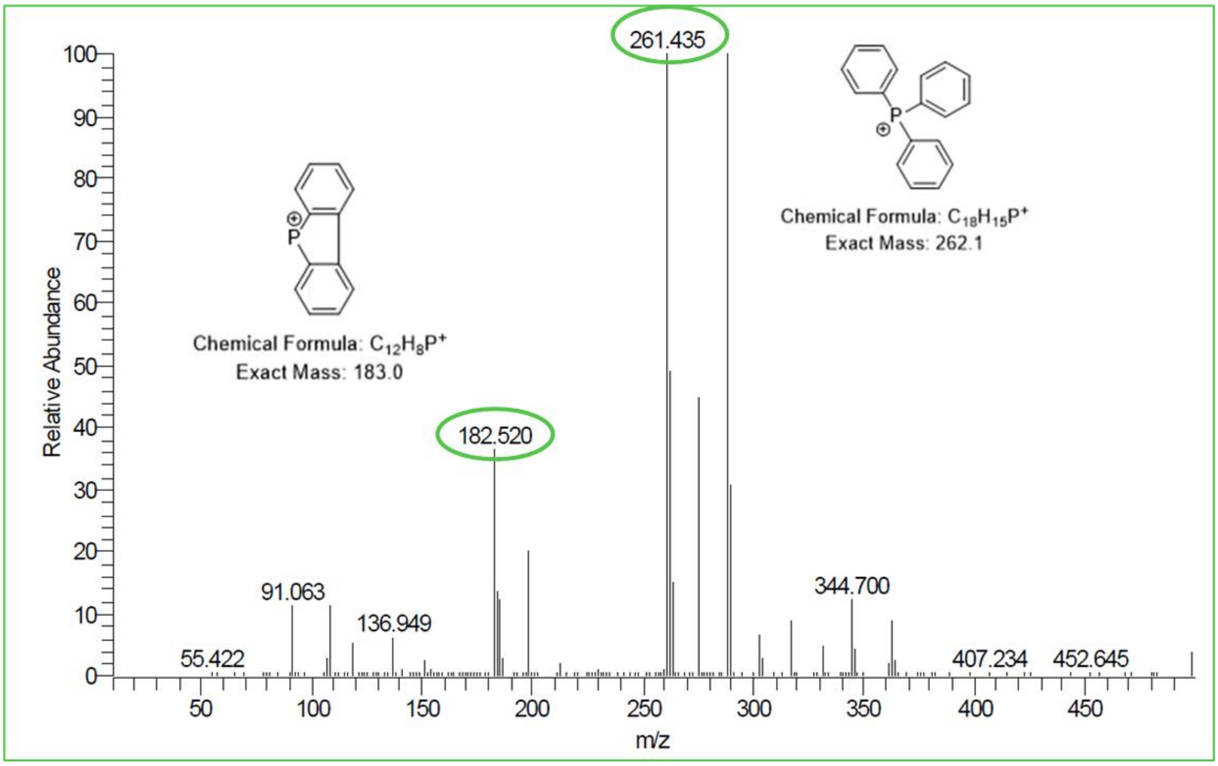

Figure S 1: MS/MS spectrum of TPP-HT and the proposed structures of the product ions.

The proposed MS/MS fragmentation mechanism of TPP-HT is illustrated in Figure S2.

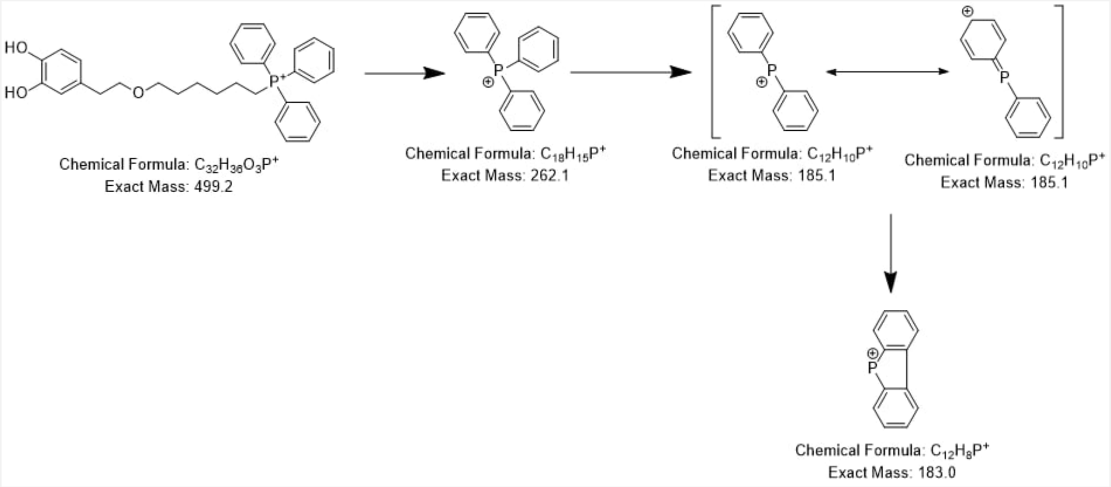

Figure S 2:Proposed MS/MS Fragmentation mechanism of TPP-HT.

The % distribution of microspecies as a function of pH is illustrated in Figure S3.

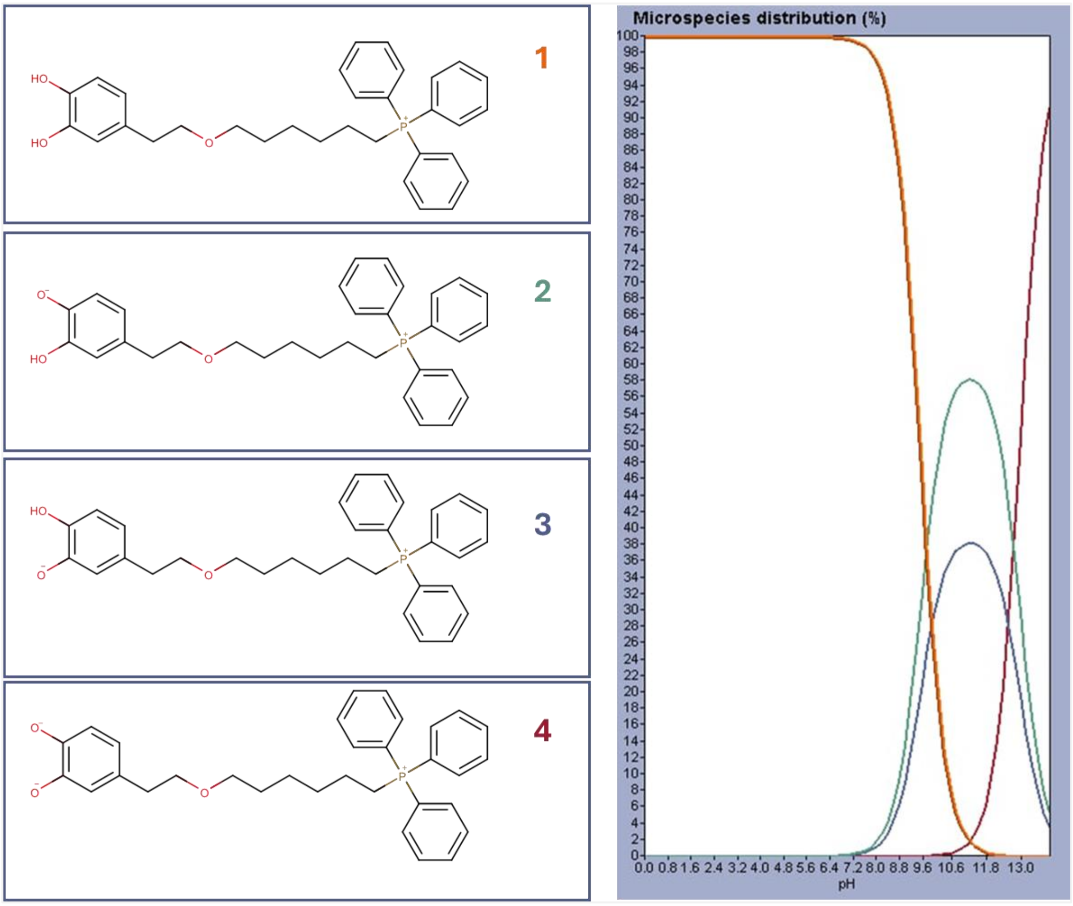

Figure S 3: Pka diagram of TPP-HT ionized microspecies as a function of pH. The orange curve corresponds to structure 1, the green curve to structure 2, the blue curve to structure 3, and the red curve to structure 4. On the left side of the panel, the structures of ionic microspecies corresponding to the four curves can be found.

The % percentage of each microspecies at different pH values is presented in Table S1.

Table S 1: %Percentage of each microspecies at different pH values.

pH	-1%	-2%	-3%	-4%
0	100	0	0	0
0.2	100	0	0	0
0.4	100	0	0	0
0.6	100	0	0	0
0.8	100	0	0	0
1	100	0	0	0
1.2	100	0	0	0
1.4	100	0	0	0
1.6	100	0	0	0
1.8	100	0	0	0
2	100	0	0	0
2.2	100	0	0	0

2.4	100	0	0	0
2.6	100	0	0	0
2.8	100	0	0	0
3	100	0	0	0
3.2	100	0	0	0
3.4	100	0	0	0
3.6	100	0	0	0
3.8	100	0	0	0
4	100	0	0	0
4.2	100	0	0	0
4.4	100	0	0	0
4.6	100	0	0	0
4.8	100	0	0	0
5	100	0	0	0
5.2	100	0	0	0
5.4	99.99	0	0	0
5.6	99.99	0.01	0	0
5.8	99.98	0.01	0.01	0
6	99.97	0.02	0.01	0
6.2	99.96	0.03	0.02	0
6.4	99.93	0.04	0.03	0
6.6	99.89	0.07	0.04	0
6.8	99.82	0.11	0.07	0
7	99.72	0.17	0.11	0
7.2	99.55	0.27	0.18	0
**7.4**	**99.29**	**0.43**	**0.28**	**0**
7.6	98.88	0.67	0.44	0
7.8	98.24	1.06	0.7	0
8	97.24	1.66	1.09	0
8.2	95.7	2.6	1.7	0
8.4	93.35	4.01	2.64	0
8.6	89.86	6.12	4.02	0
8.8	84.83	9.16	6.01	0
9	77.91	13.33	8.75	0
9.2	68.99	18.71	12.29	0.01
9.4	58.4	25.1	16.48	0.01
9.6	46.97	32	21.01	0.02
9.8	35.84	38.7	25.41	0.04
10	26.06	44.59	29.28	0.08
10.2	18.18	49.31	32.38	0.13
10.4	12.29	52.81	34.68	0.23
10.6	8.11	55.24	36.27	0.38
10.8	5.26	56.81	37.31	0.61
11	3.37	57.73	37.91	0.99
11.2	2.14	58.12	38.16	1.58
11.4	1.35	58.04	38.11	2.5

11.6	0.84	57.49	37.75	3.92
11.8	0.52	56.37	37.02	6.09
12	0.32	54.53	35.81	9.34
12.2	0.19	51.76	33.99	14.06
12.4	0.11	47.86	31.43	20.6
12.6	0.06	42.73	28.06	29.15
12.8	0.03	36.51	23.98	39.48
13	0.02	29.67	19.48	50.84
13.2	0.01	22.87	15.02	62.11
13.4	0	16.77	11.02	72.21
13.6	0	11.79	7.74	80.46
13.8	0	8.02	5.27	86.71
14	0	5.32	3.49	91.18

The logD as a function of pH for the most abundant microspecies is depicted in Figure S4.

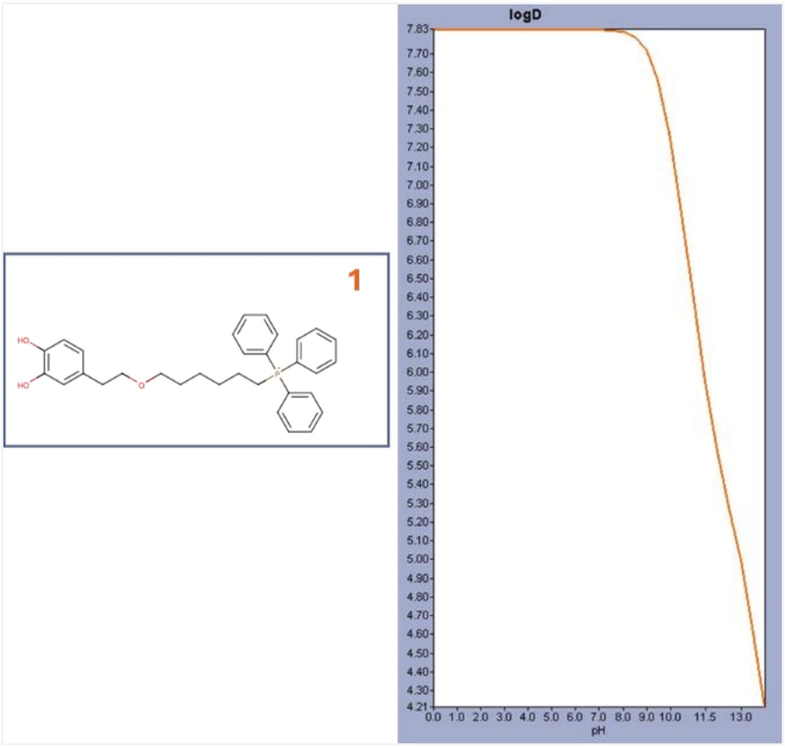

Figure S 4: logD diagram as a function of pH of the most abundant microspecies of TPP-HT under the selected experimental conditions. On the left side of the panel, the structure of the microspecies is illustrated.

The obtained logD values at distinct pH values are listed in Table S2.

Table S 2: Log D values obtained for different pH.

pH	logD
0	7.83
0.5	7.83
1	7.83
1.5	7.83
2	7.83
2.5	7.83
3	7.83
3.5	7.83
4	7.83
4.5	7.83
5	7.83
5.5	7.83
6	7.83
6.5	7.83
7	7.83
7.5	7.83
8	7.82
8.5	7.8
9	7.72
9.5	7.56
10	7.25
10.5	6.84
11	6.39
11.5	5.95
12	5.58
12.5	5.28
13	5
13.5	4.64
14	4.21

The illustration of the mesh diagram of TPP-HT using Marvin Sketch is presented in Figure S5. The logP increment is mapped on Van der Waals spheres.

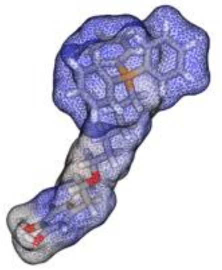

Figure S 5: Illustration of TPP-HT

Sample preparation cerebellum

In a 2 mL Eppendorf tube, 30 mg of cerebellum tissue samples were weighed, and 2 μL TPP (1 ng μL^{-1}), which was used as an IS, was added to each sample. For quantification purposes, spiking (by adding 3 μL of 0.33 ng μL^{-1} TPP-HT) was performed. The samples were left at room temperature for 1 h after spiking with the analyte and its corresponding IS to facilitate their absorption into the matrices. This was followed by the addition of 300 μL MeOH acidified with 0.01% FA and 1% ascorbic acid (serving as an antioxidant). The samples were agitated on a horizontal shaker for 15 min and centrifuged at 3000 rcf at 4 °C. The supernatants were aspirated into new Eppendorf tubes and stored at -20 °C for 16 hours. The samples were then centrifuged and the supernatants evaporated to dryness under a gentle nitrogen stream at room temperature. Finally, the residues were reconstituted by adding 0.1 mL MeOH acidified with 0.01 % FA, and the extracts were transferred to 2 mL autosampler glass vials and injected into the liquid chromatography–mass spectrometry (LC-MS/MS) system.

Chemometrics

The equations of the fortified curves of the liver and the cerebellum samples are tabulated in Tables S3 and S4, respectively.

Table S 3: Equations of the fortified curves of the liver samples.

Description	Equation of Fortified Curve	R^2
1st Day—1st replicate	$y=-(24.70 \pm 0.79)\times10^{-3}$ x-$(3.67 \pm 0 .97) \times10^{-2}$	0.996
1st Day—2nd replicate	$y=-(24.85\pm 0.76)\times10^{-3}$ x-$(3.21 \pm 0.94) \times10^{-2}$	0.996
1st Day—3rd replicate	$y=-(25.34 \pm 0.88)\times10^{-3}$ x-$(3.00 \pm 1.1) \times10^{-2}$	0.995
1st Day—consensus curve	$y=-(24.96 \pm 0.43)\times10^{-3}$ x-$(3.29 \pm 0.53) \times10^{-2}$	0.994
2nd Day—1st replicate	$y=-(18.45 \pm 0.65)\times10^{-3}$ x+$(1.43 \pm 0.68) \times10^{-2}$	0.992
2nd Day—2nd replicate	$y=-(19.30\pm 0.84)\times10^{-3}$ x+$(0.39 \pm 0.20) \times10^{-2}$	0.993
2nd Day—3rd replicate	$y=-(18.63 \pm 0.94)\times10^{-3}$ x+$(23.82 \pm 1.1) \times10^{-2}$	0.991
2nd Day—consensus curve	$y=-(18.81 \pm 40.9)\times10^{-3}$ x+$(1.38 \pm 0.48) \times10^{-2}$	0.990

Table S 4: Equations of the fortified curves of the cerebellum samples.

Description	Equation of Fortified Curve	R^2
1st Day—1st replicate	$y=(10.05 \pm 0.11)\times10^{-2}$ x-$(0.71 \pm 0.37) \times10^{-2}$	0.9995
1st Day—2nd replicate	$y=(9.97 \pm 0.22)\times10^{-2}$ x+$(2.35 \pm 1.2) \times10^{-2}$	0.998
Consensus curve	$y=(10.01 \pm 0.12)\times10^{-2}$ x-$(0.82 \pm 0.42) \times10^{-2}$	0.998

The QQ and homoscedasticity plot for cerebellum samples are illustrated in Figure S6.

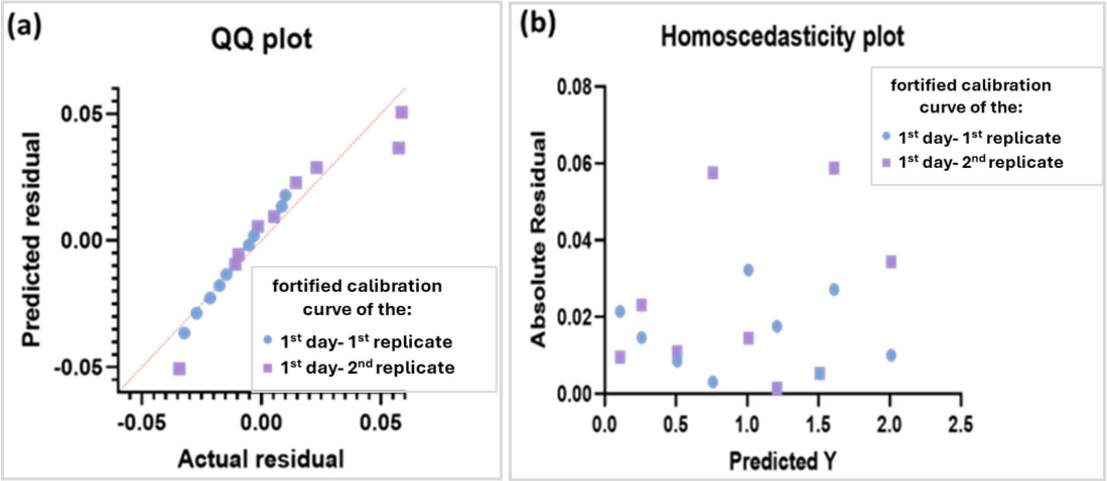

Figure S 6: (a) QQ plot of fortified curves. (b) Homoscedasticity plot of the fortified curves for cerebellum samples. The QQ plots show that the residuals are normally distributed, and the homoscedasticity plots demonstrate the homoscedasticity of the residuals.

The results of accuracy for cerebellum samples are tabulated in Table S5.

Table S 5: Results of accuracy (precision and trueness) in 5 fortification levels for cerebellum samples.

Fortification level (ng g^{-1})	Repeatability (n=6)		Intermediate precision (n=9)		Trueness (n=6)
	Average (ng g^{-1})	%RSD	Average (ng g^{-1})	%RSD	%Recovery (average ± confidence interval (95%))
2.5 (LLOQ)	2.43	3.5	2.45	3.8	103 ± 7.0
7.5 (LQC)	7.38	5.8	7.42	6.9	94 ± 11
10 (MQC)	10.2	6.4	10.4	6.8	98 ± 5.1
17.5 (HQC)	17.8	2.9	18.0	3.4	108 ± 3.4
20 (ULOQ)	19.8	5.4	19.6	5.8	102 ± 7.2

The three fortified curves of each cluster, as well as the corresponding cumulative curve for each triplicate, are illustrated in Figure S7.

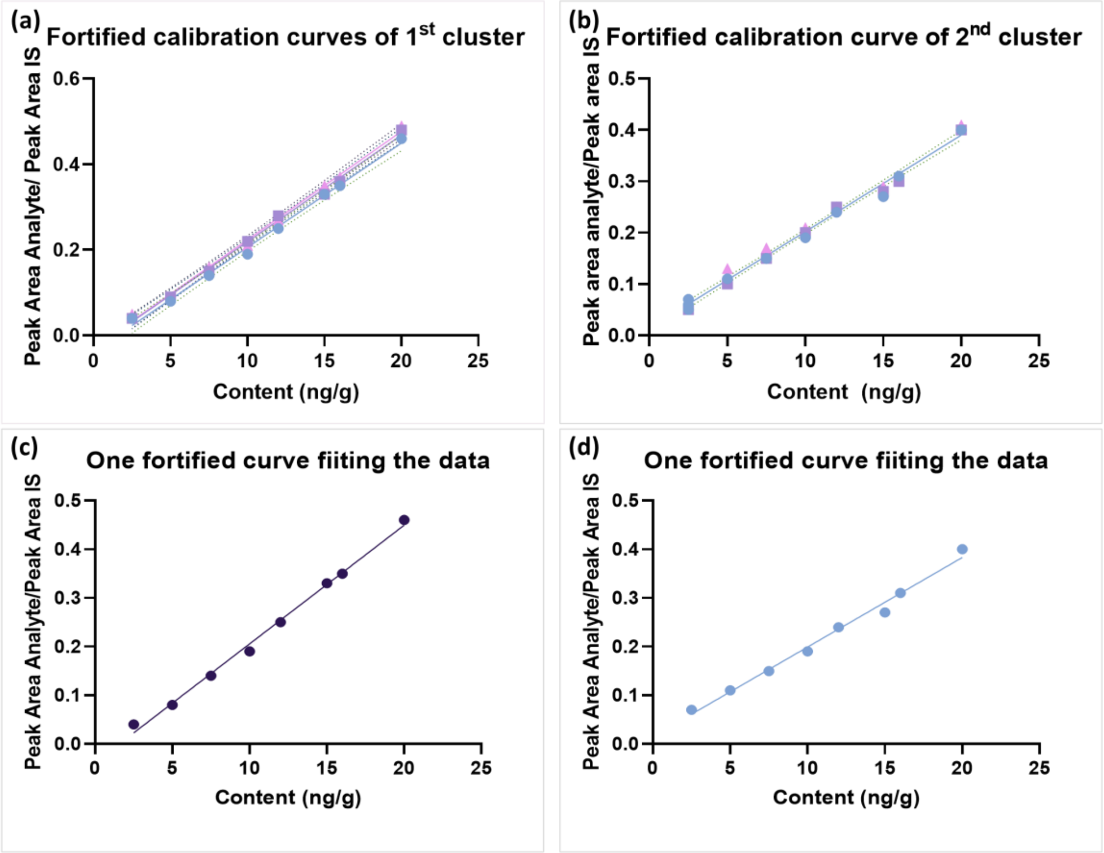

Figure S 7: (a) Fortified calibration curves of the samples of the 1st cluster. (b) Fortified calibration curves of the samples of the 2nd cluster. (c) One fortified curve fitting to the data of the 1st cluster. (d) One fortified curve fitting to the data of the 2nd cluster.
